# The Royal Victoria Hospital, Belfast

**Published:** 1901-07-06

**Authors:** 


### Editor's Letter-Box.

### THE ROYAL VICTORIA HOSPITAL, BELFAST.

Mr. W. Henman and Mr. T. Cooper, architects to the above hospital, write:—

"While thanking you for the critically appreciative article which appeared in your last issue, accompanying a plan of this building, may we ask you to afford a little space in your valuable paper in order that we may further explain a few points in connection with the subject of 'Plenum' ventilation for hospital requirements?

"You state as a drawback to the employment of the system the question of expense, and that hitherto it has been somewhat heavy; relatively it may have been so, but the fact that it is rapidly gaining favour indicates it is considered to be worth having, even at some additional cost. Nevertheless, our endeavour has been so to improve and simplify its application that on the score of expense it may favourably compare with the haphazard means more frequently employed.

"The supply of warmth in cold weather is necessarily associated with 'Plenum' ventilation; yet, as neither fireplaces, hot water, nor steam pipes are required, the first cost and up-keep for heating is decidedly economical; moreover, in this particular building, the power required for the ventilation will be secured at a minimum of expense, as it gives also a supply of hot water night and day, which is a necessity in every large hospital, and for which purpose a steam boiler of considerable capacity would under other circumstances be required to be kept going summer and winter.

"Instead of simply employing the steam for heating the water, its power will first be utilised in the engines for working the rotary air propellers, the exhaust steam being then made use of for securing the necessary hot water supply. By the employment of improved mechanical appliances, the power required for the ventilation is small, therefore the consumption of fuel need be no more for providing both power and heat than would be necessary for the hot water supply alone. In winter, when it is necessary to warm the buildings, an additional boiler is made use of, as would be the case were no mechanical means for ventilation adopted.

"As to the question whether the health and stamina of those living in an equable temperature, such as is possible with 'Plenum' ventilation, are adversely affected, there is no evidence that such is the case in buildings with which we are acquainted where the system has been properly applied; in a general hospital the patients are subject to such conditions for but a limited period, and their state of health is such that considerable variations in temperature might be prejudicial, to say nothing of the risk some might run from the action of cold draughts; and we are assured by physicians and surgeons that the natural progress of their patients can be watched with greater certainty, knowing they are not subjected to variable climatic conditions. Again, may not a rest from the wear and tear entailed in meeting the constant changes of climate to which they would ordinarily be subjected of itself facilitate the recovery of patients?

"With regard to the moot point respecting the comfort of radiant heat as compared with heat of convection, it must be remembered that very few large hospital wards depend entirely upon open fireplaces, steam or hot-water heating being employed alone or supplementarily to the open fires, simply because, as you justly say, 'in very cold weather it is quite impossible to get the full supply of air through the wards without discomfort or danger, unless means be taken to warm the air as it comes in;' consequently that pleasant state to which you afterwards refer, when the building and its contents are warmed by radiant heat, while the air remains cool, is not that of a ward naturally ventilated, but portions of the air are warmed while other portions remain cold, the result being draughts and the inrush of air from questionable sources.

"With 'Plenum' ventilation in connection with well-arranged appliances for heating, the whole body of the air supply is moderately warmed, the continuous inflow of which keeps walls and furniture at the same temperature, the condition being what would generally be described as balmy, and such as may be experienced in the shade on a calm summer's day.

"It is quite a mistake to suppose that with efficient appliances air enters the wards at a high temperature, for it rarely exceeds 65° Fahr.

"It is true that the American system of employing compact batteries of steam piping over which an inadequate supply of air is forced with an excessive expenditure of power has been introduced into this country; but such an arrangement is defective, and would never be employed by anyone with knowledge of the necessities for securing healthy ventilation and warmth.

"A further point in favour of 'Plenum' ventilation is that in hot weather wards and rooms can, without the employment of an ounce of ice, be kept continuously comfortable and cool.

"In the Belfast Hospital there will be no fear of a breakdown through the 'vagaries' of an electrical supply because the motive power will be from steam engines, with all appliances in duplicate.

"One other point of considerable importance which has become possible with the arrangement of plan adopted for the Belfast Hospital is the even distribution of air throughout the wards, there being an inlet between each bed and the next and an outlet under *every* bed.

"Trusting that these remarks will prove to those interested in the subject that, although the plan adopted for the Victoria Hospital, Belfast, is so novel in its arrangement, its development is the result of observation and experience, scientific knowledge and appliances being adopted to secure results unattainable by natural means."

